# Lilobot: A Cognitive Conversational Agent to Train Counsellors at Children’s Helplines: Design and Initial Evaluation

**DOI:** 10.1007/s10916-024-02121-8

**Published:** 2025-01-14

**Authors:** Sharon Grundmann, Mohammed Al Owayyed, Merijn Bruijnes, Ellen Vroonhof, Willem-Paul Brinkman

**Affiliations:** 1 Intelligent Systems, Delft University of Technology, Delft, The Netherlands; 2 Utrecht University School of Governance, Utrecht, The Netherlands; 3 De Kindertelefoon, Utrecht, The Netherlands; 4 College of Computer Science, King Saud University, Riyadh, Saudi Arabia

**Keywords:** Conversational agent, Chatbot, Training, Child counselling, BDI, Education

## Abstract

To equip new counsellors at a Dutch child helpline with the needed counselling skills, the helpline uses role-playing, a form of learning through simulation in which one counsellor-in-training portrays a child seeking help and the other portrays a counsellor. However, this process is time-intensive and logistically challenging-issues that a conversational agent could help address. In this paper, we propose an initial design for a computer agent that acts as a child help-seeker to be used in a role-play setting. Our agent, Lilobot, is based on a Belief-Desire-Intention (BDI) model to simulate the reasoning process of a child who is being bullied at school. Through interaction with Lilobot, counsellors-in-training can practise the Five Phase Model, a conversation strategy that underpins the helpline’s counselling principle of keeping conversations child-centred. We compared a training session with Lilobot to a text-based training, inviting experienced counsellors from the Dutch child helpline to participate in both sessions. We conducted pre- and post-measurement comparisons for both training sessions. Contrary to our expectations, the results show a decrease in counselling self-efficacy at post-measurement, particularly in Lilobot’s condition. Still, the counsellors’ qualitative feedback indicated that, with further development and refinements, they believed Lilobot could potentially serve as a useful supplementary tool for training new helpline counsellors. Our work also highlights three future research directions for training simulators in this domain: integrating emotions into the model, providing guided feedback to the counsellor, and incorporating Large Language Models (LLMs) into the conversations.

## Introduction

The Dutch Kindertelefoon is one of many child helplines worldwide that provide a safe, low-threshold, and accessible platform for young people seeking social advice and emotional support [15]. Children can reach out to the helpline through telephone or chat services regarding diverse issues, including family, relationships, sexuality, and abuse [40]. Supporting children in this manner takes practice in applying various counselling theories and conversational strategies. Typically, these are practised through role-play sessions where one counsellor-in-training (hereafter referred to as trainee) acts as a counsellor, and another portrays a child, which is useful in many settings [11, 25]. For skills acquisition, the opportunity for repeated role-playing with feedback is critical [29]. This, however, is time-intensive and logistically difficult to arrange. For instance, multiple trainees and trainers need to be physically present at the same location for maximum effect. Using an interactive computer agent could prove useful to overcome these limitations [35], as it provides a safe, affordable, and accessible environment [13, 30].

In this paper, we present a conversational agent that simulates a child help-seeker for training new counsellors at child helplines. Through interaction with the agent, a trainee could learn to apply the Five Phase Model [38], a conversation strategy often used by helplines. The conversational strategy supports the dynamics of a conversation while ensuring that the conversation remains child-centred. The Five Phase Model starts by (1) building rapport with the child, (2) clarifying the child’s story, (3) setting the session’s goal, (4) working toward the goal, and finally (5) rounding off the conversation, with each phase having guidelines to move the conversation forward successfully. As pointed out earlier, conversational agents designed for training communication skills can provide a safe learning environment, facilitate the development of communication skills [21], and enhance students’ self-efficacy [37]. Such agents include Bruijnes’ virtual crime suspect for training interrogation skills [6] and virtual patients in the medical domain [7, 9]. However, limited work has been done using agents to train new helpline counsellors. An exception is the work of Demasi et al. [10], who proposed a conversational agent for training suicide prevention hotline counsellors. They found differences in evaluation between counsellors and crowdsourced workers, with counsellors rating the agent’s dialogue as less coherent, useful, and consistent. This highlights the importance of clearly defining and involving the target group in agent evaluations.

## Design of Conversation Simulator

To explore the potential of using a conversational agent as a learning tool, we took a conversation about bullying as a scenario for trainees to learn how to apply the Five Phase Model. We developed Lilobot, a conversational agent, i.e., chatbot, that simulates a child who is being physically bullied at school and reaches out to a children’s helpline via their chat-based textual interface (Fig. 7). This agent mimics the interactions of such interfaces. Lilobot was intended to be gender-neutral. We designed Lilobot’s simulated child help-seeker scenario together with experts from the helpline, ensuring the agent exhibits behaviours associated with victims of school bullying such as low self-esteem and loneliness [3], social anxiety [19], and poor academic performance [12]. The trainee’s objective is to counsel Lilobot according to the Five Phase Model so that Lilobot is encouraged to seek help from people in its network, such as parents or teachers (a confidant). We set the initial beliefs and desires held by Lilobot in a way that requires trainees to apply the Five Phase Model to complete the conversation successfully. For that, we intentionally included beliefs that defied common expectations, like Lilobot suggesting the helpline contact the school. As the helpline should not do this, it allows trainees to experience the benefits of following guidelines and what could happen if, for example, they spend too little time clarifying the child’s story.

### Lilobot Thinking

Lilobot’s ‘thinking’ process is based on a Belief-Desire-Intention (BDI) computational model which forms the foundation of its decision-making. In simple terms, Lilobot holds various beliefs about itself and the world which are affected by the trainee’s input during the conversation. The beliefs function as parameters on an interval scale ranging from 0 to 1 that can be set at the start, and these values can increase or decrease during conversation based on the trainee’s input. By varying the initial parameter settings, trainees can practice with different configurations of Lilobot, who holds different beliefs and desires and, therefore, reacts differently in a conversation. For convenience, we worked with one fixed initial parameter setting, which formed its beliefs and desires, i.e., the conversational goals it wanted to achieve. Its beliefs and desires determine its intention, which is the desire that is most prominent at the point in the conversation. Through this intention, Lilobot decides on an appropriate response to the trainee, i.e., an action it selects from its knowledge base. Lilobot’s desires represent the child’s goals held at a specific moment in the conversation. The intentions align with how children might respond if a counsellor follows or deviates from these phases.

**Fig. 1:**
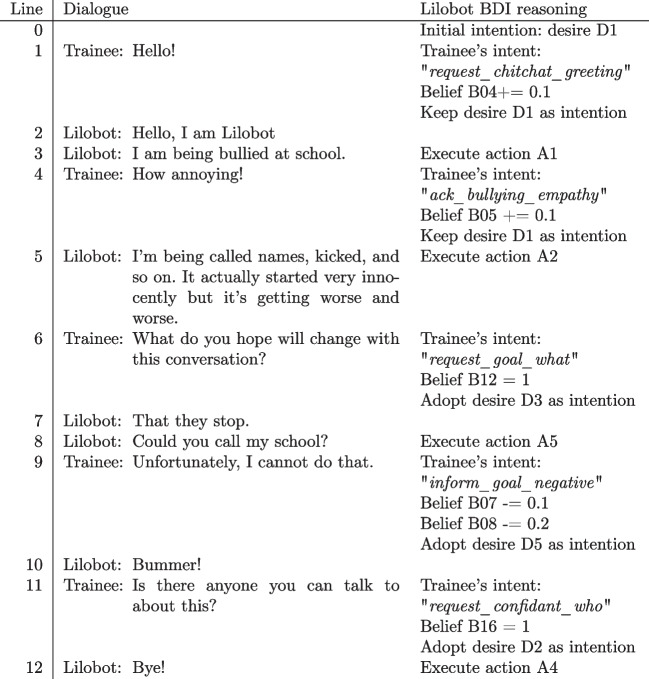
Sample conversation with Lilobot and corresponding BDI reasoning. In this scenario, the trainee rushes through the second phase of the Five Phase Model, resulting in Lilobot leaving the conversation later on. Lilobot’s beliefs, desires, and intentions are listed in Tables 1, 2, and 3, respectively

Let us illustrate the interactions between Lilobot and a trainee through a scenario depicted in Fig. 1. The scenario ends in an undesired outcome as the trainee rushes through the second phase instead of asking questions to explore the problem and also showing empathy. When the trainee types their input, Lilobot uses its Natural Language Understanding (NLU) model to classify it. In line 1, the input is recognised as a greeting to initiate the conversation, which increases Lilobot’s belief (B04) that the helpline can be trusted. Lilobot’s current desire (D1: Lilobot wants to talk about its problem) remains unchanged as the belief value thresholds for the subsequent desire have not been met. Therefore, Lilobot proceeds with the next action (A1) linked to the desire D1, which is to introduce the problem. When the trainee expresses empathy towards the child’s situation (line 4), Lilobot’s belief that the trainee understands it (B05) increases. As a result, Lilobot provides more information about the problem (action A2; linked to D1). When asked about its goal, Lilobot increases its belief that the trainee asks about its wish (B12), consequently shifting its desire to D3, which expresses its wish for the helpline to get the bullies out of school. Lilobot then responds with its goal beyond the scope of this conversation (line 7) and responds with a goal that the helpline cannot fulfil-asking the helpline to contact its school (line 8; A5). As mentioned earlier, this is a rather uncommon request, but we included it to show the importance of adhering to the Five Phase Model. The trainee makes another mistake by rejecting the request to call the school, without suggesting what the child can do instead (line 9). Thus, Lilobot’s beliefs about the trainee’s ability to solve the problem (B07 and B08) decrease. As the trainee rushes through the second phase, Lilobot’s beliefs that the trainee understands its story (B05) and is interested in it (B06) decreases. Therefore, Lilobot begins to doubt the trainee’s ability to help, subsequently shifting its desire to D2, which is to end the conversation. Consequently, Lilobot executes its action by saying “Bye!” (A4; linked to D2). Tables 1, 2, and 3 respectively show lists of all beliefs, desires, and actions that Lilobot holds.

Another feature of the agent is that it can initiate a conversation if the trainee has not sent any messages for 10 seconds. In such cases, Lilobot responds with a message related to its current desire. To achieve this, Lilobot retrieves the next incomplete action that is linked to the desire and uses that as a response. This behaviour is demonstrated in lines 3 and 5 of the dialogue (Fig. 1), where Lilobot discusses the issue of bullying.

As giving feedback is critical for skills acquisition [29], the training tool provides a transcript of the conversation and Lilobot’s starting and ending beliefs after the conversation (shown in Table 1). The feedback also indicates the relevance of each belief to the phases of the Five Phase Model. Moreover, it shows how beliefs change during the conversation, with a positive number indicating that Lilobot held a more desirable belief at the end of the conversation than at the start, from the helpline’s perspective.

**Table 1:** Beliefs of the conversational agent Lilobot and their relation to the Five Phase Model phases

ID	Belief	Phase	$\Delta$
*About self*			
B01	Lilobot thinks it is in control	All	0
B02	Lilobot thinks it is competent to solve the problem	4	0
B03	Lilobot feels connected to the child helpline	All	0
*About the child helpline trainee*			
B04	Lilobot thinks the trainee can be trusted	2	0.1
B05	Lilobot thinks the trainee understands it	All	0.1
B06	Lilobot thinks the trainee is interested in its story	2	0
B07	Lilobot thinks the trainee can help it	3	−0.1
B08	Lilobot thinks the trainee can solve its problem	3	−0.2
B09	Lilobot thinks it and the trainee will be able to reach a solution	4	0
B10	Lilobot thinks the trainee is going to solve its problem	4	0
*About conversation*			
B11	Lilobot thinks it has talked about its situation	2	0
B12	Lilobot thinks the trainee is asking about its wish	3	1
B13	Lilobot thinks the trainee is asking about a positive wish	3	0
B14	Lilobot feels safe in the conversation	All	0
B15	Lilobot thinks the trainee wants to end the conversation	5	0
*About confidant*			
B16	Lilobot thinks the trainee is asking about a confidant	4	1
B17	Lilobot thinks its teacher can help it	4	0

The sample feedback shows belief value differences ($\Delta$) between the end and start of the conversation in Fig. 1

**Table 2:** Desires of the conversational agent Lilobot and the corresponding phase of the Five Phase Model

ID	Desire name	Phase
D1	Lilobot wants to talk to about its problem	Phase 2
D2	Lilobot wants to end the conversation	Phase 5
D3	Lilobot wants the trainee to get the bullies out of school	Phase 3
D4	Lilobot wants to talk to its teacher about its problem	Phase 4
D5	Lilobot wants to work with the trainee to find a solution	Phase 3

**Table 3:** List of actions that Lilobot performs and their corresponding desire, mentioned in Table 2

ID	Action name	Desire
A1	Lilobot introduces the problem	D1
A2	Lilobot provides more details about the problem	D1
A3	Lilobot talks about the emotional impact of the problem	D1
A4	Lilobot says goodbye	D2
A5	Lilobot asks the trainee to call the school to get the bullies out	D3
A6	Lilobot asks how teacher can help it	D4
A7	Lilobot expresses concern about the bullying getting worse	D4
A8	Lilobot asks the trainee what to tell its teacher	D4

**Fig. 2:**
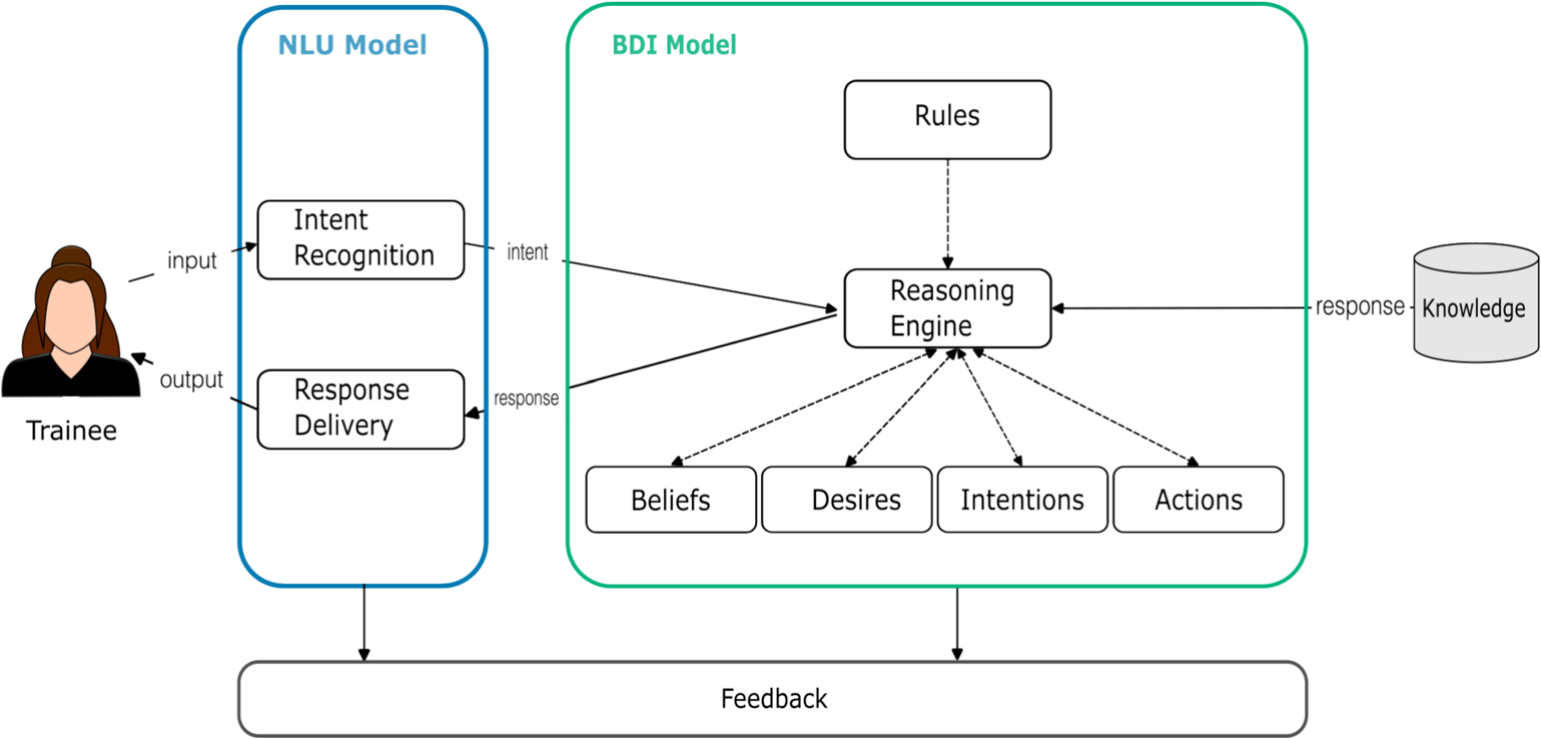
Architecture of Lilobot

### Lilobot’s Architecture

To provide a comprehensive understanding of the Lilobot agent’s design, Fig. 2 displays the architecture of the system. It includes seven main components explained in Table 4 below.

**Table 4:** Components of Lilobot

Component	Description
Intent recognition	The agent first classifies the trainee’s raw text message input using a pre-trained NLU model. This intent classification is then sent to the BDI model to reason about.
Reasoning engine	Based on the trainee’s intent that it receives, the reasoning engine updates the beliefs, desires, and actions of the agent. It is responsible for selecting an intention for the agent, based on a defined set of rules.
Rules	This is a mapping of values that determines by how much a desire or belief is updated given a trainee’s intent and the resulting intention.
Beliefs	We modelled 17 beliefs (shown in Table 1) as statements that have a value ranging from 0 to 1 (weak to strong). These beliefs are split into subgroups - those about the conversation, the child helpline (trainee), the people in the child’s network (confidant), and the simulated child.
Desires	We designed five desires that the agent could seek to accomplish during a conversation with a trainee (Table 2). Desires have state values that indicate whether they are active or not. Similar to beliefs, these are updated during the conversation.
Intentions	An intention is a desire marked active and is currently being pursued by the agent. They are updated throughout the conversation.
Actions	For each desire, we defined an action or a sequence of actions that the agent executes to achieve the desire (Table 3). Each action has a binary value indicating whether or not it has been completed.
Response delivery	This component of the NLU model returns the response from the BDI model to the trainee.

## Evaluation

### Method

The experiment had a within-subject design with two conditions: text-based intervention as a simple text explaining the Five Phase Model, and the conversational agent (Lilobot), our interaction-based intervention. We evaluated Lilobot using four measures: (1) trainees’ self-efficacy in applying the Five Phase Model, (2) their perceived usefulness of the learning tool, (3) system usability, and (4) the conversation’s outcome (i.e., Lilobot’s end belief values). We also collected qualitative data through five open-ended questions to gain insight into the participants’ experiences. In total, we invited 39 counselling volunteers from the Dutch child helpline to participate in the experiment through email. We used a counterbalanced design to control for order effects. For this, we split participants into two groups, where each group experienced both interventions but in reverse order. After excluding 11 participants for not completing the questionnaires, we had a total of 28 helpline counsellors with varying years of counselling experience ranging from 0 to 16 years (*M* = 3.54 years, *SD* = 3.95). We asked the participants to complete all questionnaires through the Qualtrics platform. Seven participants did not complete all self-efficacy questions. For six of them, we calculated the average score based on the items they had answered, and one was excluded from the self-efficacy analysis as this person had not provided any responses. As for the outcome of the conversation, we calculated the average belief values held by the agent at the end of a session.

We requested the participants to complete the experiment in one sitting, taking about an hour. They signed an informed consent form and completed a pre-training questionnaire about their counselling experience at the helpline and initial counselling self-efficacy measurements. This was followed by the two training interventions. After each intervention, participants completed questionnaires on their counselling self-efficacy, inspired by established measures [1, 26], and checked by supervisors at the children’s helpline. The questionnaire included eight items ranging from -5 ‘strongly disagree’, 0 ‘neutral’ to +5 ‘strongly agree’, for which we analysed the mean. During the intervention with Lilobot, participants engaged with the agent in three consecutive sessions, each lasting approximately 15 minutes. The goal of the first and third sessions was to counsel Lilobot according to the Five Phase Model, while the second session allowed participants to explore the agent. After each session with Lilobot, the agent provided feedback based on the BDI status of the simulated child help-seeker. Upon completing the study, participants rated Lilobot’s perceived usefulness on eight items ranging from -5 ‘negative’ to +5 ‘positive’, with 0 indicating neutral. These items, adapted from previous research [17, 27, 39], were analysed separately. The participants also filled out the usability questionnaire, which was a Dutch version of the System Usability Scale (SUS) questionnaire [5] containing ten items [20, 41]. Each item was rated on a 5-point scale from 0 ‘strongly disagree’ to 4 ‘strongly agree’. To calculate an interpretive score out of 100, we reversed the score of four reverse wording questionnaire items and summed the scores of all ten items, then multiplied the score by 2.5. For the analysis, we conducted a repeated measures ANOVA on the self-efficacy data to evaluate the main effect and an interaction effect of the two independent variables - the training intervention and the time of measurement (e.g., before or after the specific training). For the remaining analyses, we used a one-sample Wilcoxon signed-rank for perceived usefulness and a paired sample *t*-test on the conversational outcome.

We analysed the responses to the three open questions through a thematic analysis [4], and used double-coding to check the reliability of the themes. The first author, with a background in computer science and artificial intelligence, identified the themes and the related coding scheme, which a second coder, a computer science graduate student, used to code responses independently. Beforehand, the second coder was trained on synthetic data generated by ChatGPT. The inter-reliability between the two coders showed a substantial level of agreement for the first (Cohen’s $\kappa$ = 0.63) and third (Cohen’s $\kappa$ = 0.68) qualitative questions, and moderate agreement for the second (Cohen’s $\kappa$ = 0.52), according to Landis and Koch [23]^1^. The coders discussed cases of disagreements to reach a consensus.

The experiment was approved by the TU Delft Human Ethics Research Committee (HREC reference number: 1622), and its design was pre-registered on the Open Science Framework (OSF) ahead of data collection^2^. All statistical analyses were done using R software (version 4.1.2). The questionnaires, dataset and the analysis R-script are available online through the 4TU research data repository.^3^

**Fig. 3:**
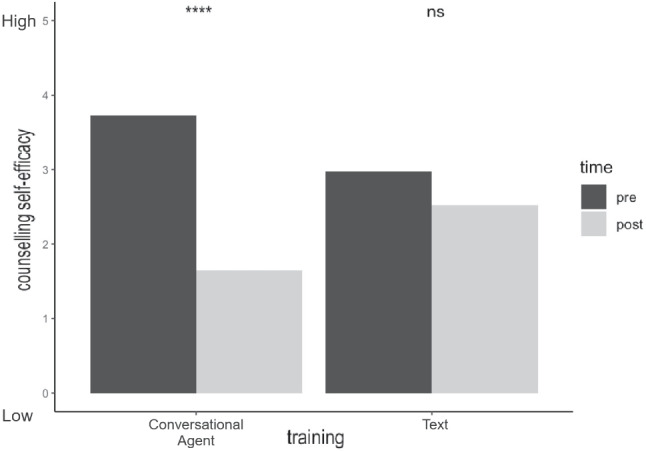
Comparing participants’ counselling self-efficacy across the text and conversational agent training interventions before and after training

### Results

#### Quantitative Results

The analysis revealed no significant main effect on counselling self-efficacy based on the type of intervention (*F*(1, 78) = 0.2, *p* = .65). However, we observed a significant main effect at different times of measurement (*F*(1, 78) = 17.32, *p*< .001), where post-counselling self-efficacy (*M* = 2.16, *SD* = 2.39) was lower than pre-counselling self-efficacy (*M* = 3.4, *SD* = 1.44). The analysis also found a significant two-way interaction effect (*F*(1, 78) = 6.52, *p* = .01) between these two variables. A follow-up simple effect analysis revealed a significant difference (*t*(78) = 4.75, *p*< .001) in counselling self-efficacy before (*M* = 3.72, *SD* = 0.93) and after (*M* = 1.71, *SD* = 2.61) training for the conversational agent intervention, but no significant effect was found (*t*(78) = 1.14, *p* = .26) in the text intervention across the two time points of measurement (Fig. 3).

**Fig. 4:**
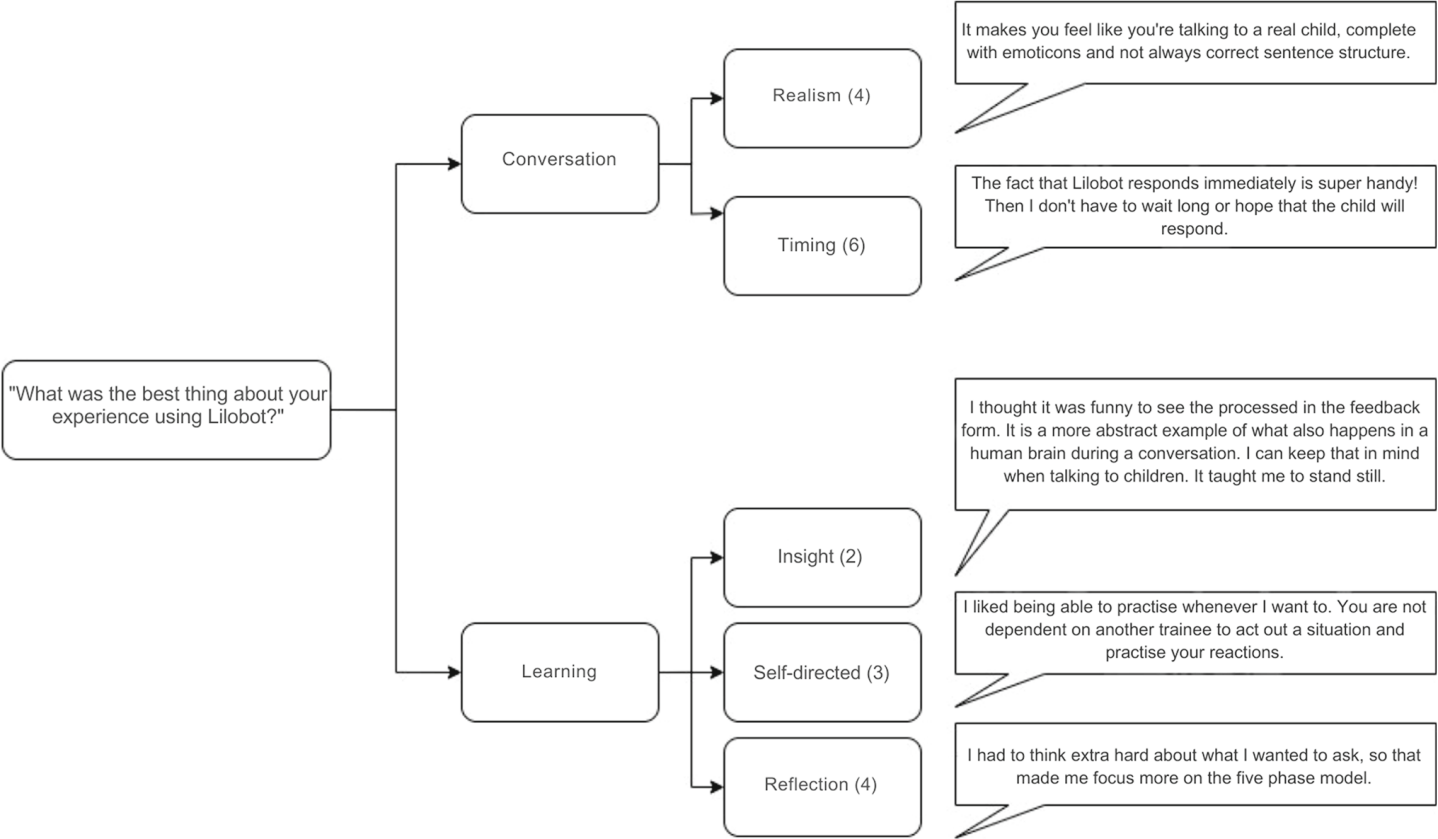
Thematic map of participants’ most liked features about their experience of using Lilobot

In our analysis of Lilobot’s perceived usefulness, participants’ ratings deviated from the neutral zero in two out of the eight items. Specifically, mean ratings were negative for participants’ self-efficacy concerning the Five Phase Model (*M* = -1.06, *SD* = 1.71 *Z* = -1.98, *p* = .02), and the usefulness of conversational agents as a learning tool (*M* = -1.62, *SD* = 2.56, *Z* = -2.29, *p* = .01). For usability, we report an average score of 67 (*SD* = 6.44), which can be interpreted as “ok” based on an adjective rating scale for the SUS questionnaire by Bangor et al. [2]. For the conversational outcome, a paired sample *t*-test showed no significant difference in the model’s conversational outcome (*t*(25) = -1.72, *p* = .1) of the first session interacting with Lilobot (*M* = 6.36, *SD* = 1.36) compared to the third session (*M* = 6.68, *SD* = 1.24).

#### Qualitative Results

The analysis identified two main themes for the question “What was the best thing about your experience using Lilobot?”: the conversation with Lilobot and the learning experience obtained from the interaction. Some participants liked that the conversation realistically simulated a child’s language style and behaviour (*n* = 4, 14%). Others appreciated the fast response time of the agent (*n* = 6, 21%). Regarding learning, participants indicated that through their experience with Lilobot, they could reflect on what they said and the Five Phase Model (*n* = 4, 14%) and see how their actions affected the agent’s behaviour (*n* = 2, 7%). Participants also noted the opportunity for self-directed learning with Lilobot as they did not have to depend on the involvement of other participants to role-play (*n* = 3, 10%). Figure 4 shows a thematic map of these responses.

**Fig. 5:**
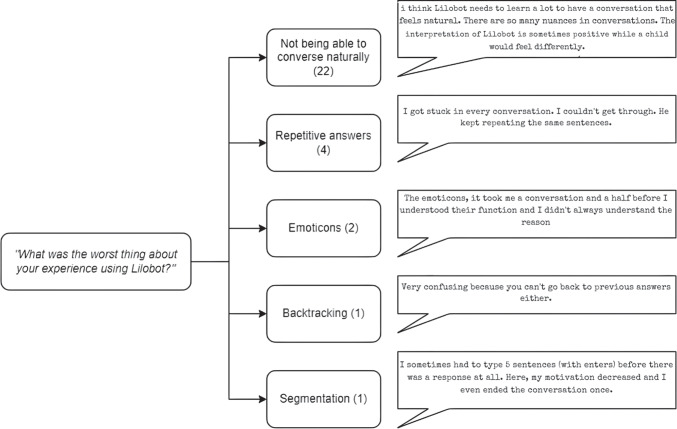
Thematic map of participants’ least liked features about their experience of using Lilobot

Figure 5 shows a thematic map of participants’ responses to the question “What was the worst thing about your experience using Lilobot?”. The most common theme identified was issues related to Lilobot’s understanding which made it difficult to hold a natural conversation (*n* = 22, 79%). Participants indicated that Lilobot did not understand their utterances or gave no response to questions they posed to the agent. Others also mentioned they received repetitive answers (*n* = 4, 14%), had difficulties understanding Lilobot’s use of emoticons (*n* = 2, 7%) and found the segmentation of utterances demotivating (*n* = 1, 4%).

**Fig. 6:**
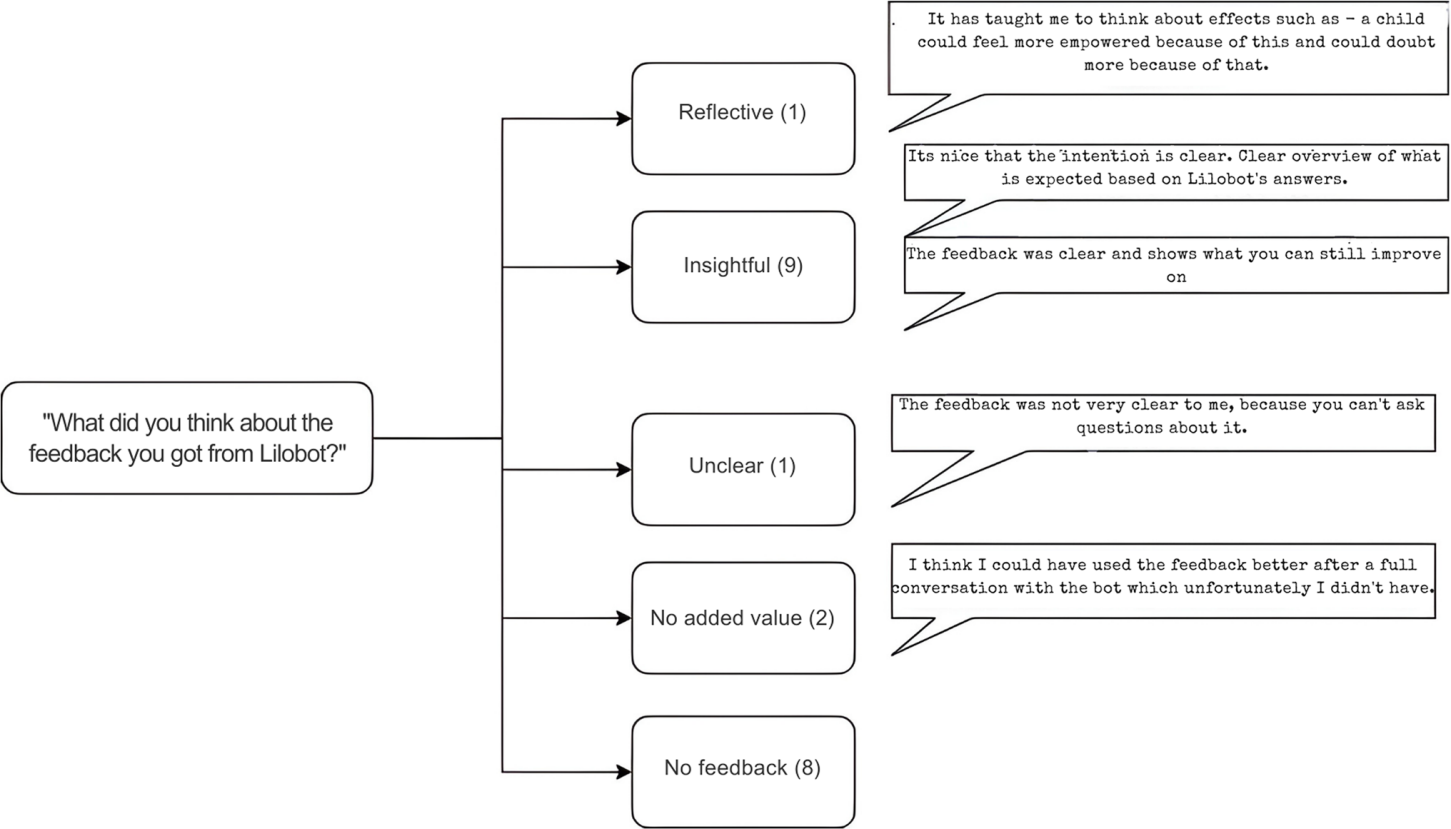
Thematic map of participants’ positive and negative remarks on feedback from Lilobot

**Fig. 7:**
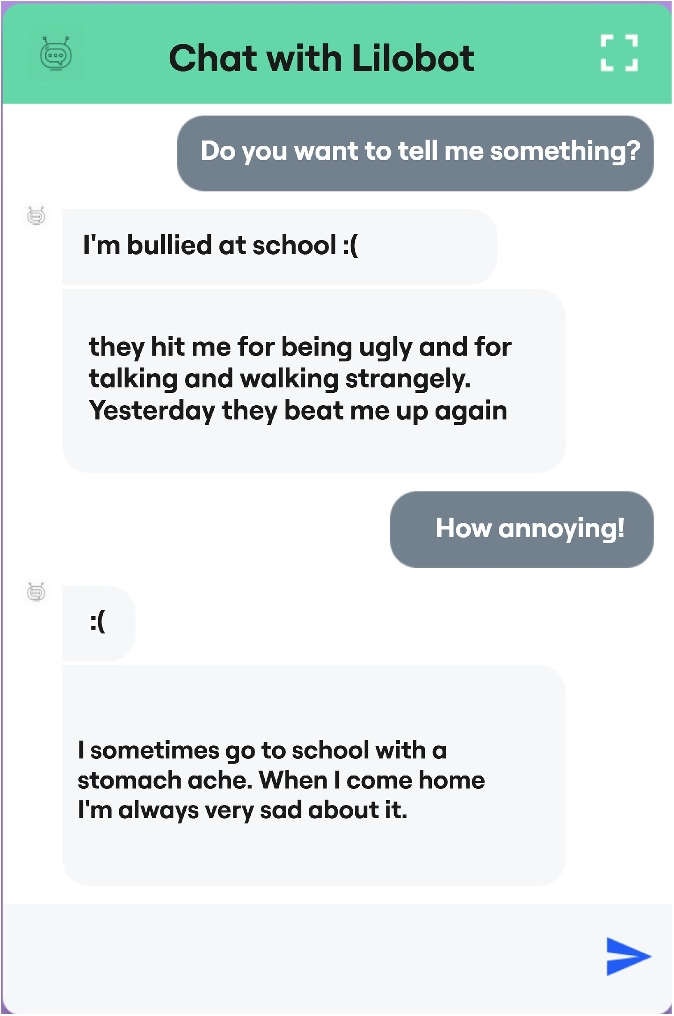
A modified screenshot of Lilobot’s interface, with messages translated from the original Dutch text

We also asked the participants about the feedback given by Lilobot. Eight out of the 28 stated they did not receive any feedback. Some participants found it insightful to see Lilobot’s reasoning process and how their actions influenced the agent’s responses (*n* = 9, 32%). On the other hand, some participants noted the feedback was of little value to them (*n* = 2, 7%), as they could not proceed in the scenario. Figure 6 shows a thematic map of participants’ responses to this question.

The final question was about which group of users the participants were likely to recommend Lilobot to. The options included counsellors-in-training (*n* = 17, 61%), novice counsellors (*n* = 3, 11%), experienced counsellors (*n* = 3, 11%), and supervisors of the helpline (*n* = 0, 0%). For the counsellors-in-training at the helpline, one reason given was that it would allow them to experiment and gain familiarity with the conversation model without real-life consequences if they did something wrong. Other participants suggested that the conversational agent might be more suited for experienced counsellors, who already understand how children behave and could use it as an opportunity to revise question-answering techniques and how they relate to the phases of the conversation model.

## Discussion and Conclusion

The experience with the conversational agent led to a decrease in the trainees’ self-efficacy. This might mean that interaction with the agent needs improvement, or that our participants scaled down their initial overestimation of their self-efficacy. As the participants were experienced counsellors, a Dunning-Kruger cognitive bias, i.e., overestimation caused by limited experience, seems less likely at play here [22]. Moreover, participants might have focused specifically on self-efficacy towards counselling an agent (Lilobot), instead of indicating their counselling self-efficacy toward any child. Still, lower self-efficacy could be beneficial, as Nissen-Lie et al. [33] found that therapists with higher self-doubt produce better therapeutic outcomes, potentially indicating a higher quality of counselling in this evaluation.

Participants perceived the usefulness of this agent as a learning tool as negative. However, from the open questionnaires, an extensive group was still positive about using the conversational agent as an additional learning opportunity for trainees, e.g., to apply theoretical knowledge in a chat situation, though not in Lilobot’s current form. They noted Lilobot’s understanding of questions needed improvement, and the responses needed more variability. This issue was caused by Lilobot misclassifying or not recognising utterances, which was apparent when participants used complex sentence structures (e.g., paraphrases) and when they delved deeper into Lilobot’s story. This was due to the limited knowledge captured in the agent’s knowledge base and the lack of segmentation of user input. On the other hand, some participants thought the conversational agent might hinder trainees from developing their own counselling style, given the agent’s simplistic question-and-answering style. All these issues arose due to the agent’s limitation of assigning only single, not multiple, classifications to a trainee’s input. Anticipating some problems, we instructed participants at the start of the experiment to separate their thoughts into separate input turns. Reflecting on the feedback, including instructions for trainees on improving their performance based on the agent’s BDI status would be more helpful than just reporting the BDI changes. Trainees need to recognise and locate their mistakes, understand and analyse them appropriately, and then take some corrective action [8, 32]. This is supported by Salmi et al. [36] work on support systems for counsellors, where they argue that counsellors value short, actionable information that is highly accurate to the situation and preferably given by someone with expertise.

Furthermore, various helpline conversational strategies clashed in our setting, such as the Five Phase Model and Setting Limits on children’s inappropriate behaviour in a conversation. Designers need to be aware of this. For example, we observed a common pattern where Lilobot repeatedly mentioned not being able to understand the question, or persisting in its request to have the trainee call the school. In these cases, the trainee would end the conversation, as they are trained to set boundaries and encourage the child to reach out again when they are ready to cooperate. This raises the question of whether strategies like the Five Phase Model can be practised independently of other counselling strategies or whether this issue mainly arises with more experienced counsellors who have been exposed to multiple strategies. On the other hand, we should be cautious about generalising the findings from the experienced counsellors to new trainees, as they might experience Lilobot differently.

## Future Research Directions

We built the BDI-based conversational agent to simulate a child help seeker and engage trainees to adhere to the Five Phase Model and its underlying guidelines. The majority of the participants, however, reported a decrease in self-efficacy. Despite the tool not being ready in its current form, we believe the results warrant further research because of its potential. From our findings, we see four directions for future research. Firstly, understanding emotions is a key part of counselling sessions [24], which justifies research into incorporating emotional aspects into this BDI model [28]. This would simulate the interplay between the trainee’s inputs on the child’s emotions and the influence of the child’s emotional intensity on their behaviour. Emotions were successfully incorporated with BDI in other contexts [16, 34]. Secondly, enriching the training system with real-time feedback, providing guidance and feedback during interactions, might be worthwhile. Currently, the system only provides a feedback summary post-session. Thirdly, expanding Lilobot to include a wider variety of cases would prepare trainees for the range of topics children might seek advice on, such as relationships, sexuality, and study issues [40]. Lastly, Lilobot’s reasoning was built on a rule-based model. With the advent of Large Language Models (LLMs) [18], a future outlook might be to incorporate such models in this setting to improve response generation.

## Data Availability

All statistical analyses were done using R software (version 4.1.2). The dataset and the analysis R-script are available online through the 4TU research data repository. https://data.4tu.nl/datasets/7b024697-659a-47ad-95a4-0497bf52b432

## Footnotes

1 Note that McHugh [31] would categorize the agreement for the first and third questions as moderate, and for the second as weak.

2 https://osf.io/hkxzc

3 https://data.4tu.nl/datasets/7b024697-659a-47ad-95a4-0497bf52b432

## References

[1] Axboe, M.K., Christensen, K.S., Kofoed, P.E., et al., Development and validation of a self-efficacy questionnaire (se-12) measuring the clinical communication skills of health care professionals. *BMC Med. Educ. *16(1):1–10, 2016. 10.1186/s12909-016-0798-7 PMC5069791 27756291

[2] Bangor, A., Kortum, P., and Miller, J., Determining what individual sus scores mean: Adding an adjective rating scale. *J. Usability Stud.* 4(3):114–123, 2009.

[3] Boivin, M., and Hymel, S., Peer experiences and social self-perceptions: a sequential model. *Dev. Psychol.* 33(1):135, 1997. 10.1037//0012-1649.33.1.135 9050398

[4] Braun, V., and Clarke, V., Using thematic analysis in psychology. *Qual. Res. Psychol.* 3(2):77–101, 2006.

[5] Brooke, J., et al., Sus-a quick and dirty usability scale. *Usability Evaluation in Industry* 189(194):4–7, 1996.

[6] Bruijnes, M., *Believable suspect agents: response and interpersonal style selection for an artificial suspect*. PhD thesis, University of Twente, 2016.

[7] Bruijnes, M., Kesteloo, M., and Brinkman, W.P., Reducing social diabetes distress with a conversational agent support system: a three-week technology feasibility evaluation. *Front. Digit. Health* 5:1149374, 2023. 10.3389/fdgth.2023.1149374 PMC10294428 37383944

[8] Carroll, J., Minimalist design for active users. In: *Unknown Host Publication Title*. North-Holland, p 39–44, 1985.

[9] Consorti, F., Mancuso, R., Nocioni, M., et al., Efficacy of virtual patients in medical education: A meta-analysis of randomized studies. *Comput. Educ.* 59(3):1001–1008, 2012.

[10] Demasi, O., Li, Y., and Yu, Z., A multi-persona chatbot for hotline counselor training. In: *Findings of the Association for Computational Linguistics: EMNLP 2020*, pp 3623–3636, 2020.

[11] Froehle, T.C., Robinson, S.E., and De Kurpius, W.J., Enhancing the effects of modeling through role-play practice. *Couns. Educ. Superv.* 22(3):197–206, 1983.

[12] Glew, G.M., Fan, M.Y., Katon, W., et al., Bullying, psychosocial adjustment, and academic performance in elementary school. *Arch. Pediatr. Adolesc. Med.*159(11):1026–1031, 2005. 10.1001/archpedi.159.11.1026 16275791

[13] Gratch, J., DeVault, D., and Lucas, G., The benefits of virtual humans for teaching negotiation. In: *International Conference on Intelligent Virtual Agents*, Springer, pp. 283–294, 2016.

[14] Grundmann, S., *A bdi-based virtual agent for training child helpline counsellors*, 2022.

[15] International, C.H., Voices of children and young people in the eu 2018, 2018.

[16] Jiang, H., Vidal, J.M., and Huhns, M.N., Ebdi: an architecture for emotional agents. In: *Proceedings of the 6th international joint conference on Autonomous agents and multiagent systems*, pp 1–3, 2007.

[17] Kang, N., Public speaking in virtual reality: Audience design and speaker experiences. *Dissertation (tu delft), Delft University of Technology*, 2016. 10.4233/uuid:e920dec8-2b71-4377-bc0f-8f9c950fff42.

[18] Kasneci, E., Seßler, K., Küchemann, S., et al., Chatgpt for good? on opportunities and challenges of large language models for education. *Learn. Individ. Differ.* 103:102274, 2023.

[19] Klomek, A.B., Marrocco, F., Kleinman, M., et al., Bullying, depression, and suicidality in adolescents. *J. Am. Acad. Child Adolesc. Psychiatry* 46(1):40–49, 2007. 10.1097/01.chi.0000242237.84925.18 17195728

[20] Koning, D., *Ontwerp van een online zelf-assessment voor het meten van de fysieke activiteit bij ouderen tussen de 55 en 75 jaar*. Master’s thesis, University of Twente, 2016.

[21] Kononowicz, A.A, Woodham, L.A., Edelbring, S., et al., Virtual patient simulations in health professions education: systematic review and meta-analysis by the digital health education collaboration. *J. Med. Int. Res.* 21(7):e14676, 2019. 10.2196/14676 PMC6632099 31267981

[22] Kruger, J., and Dunning, D., Unskilled and unaware of it: how difficulties in recognizing one’s own incompetence lead to inflated self-assessments. *J. Person. Social Psychol.* 77(6):1121, 1999. 10.1037//0022-3514.77.6.1121 10626367

[23] Landis, J.R., and Koch, G.G., The measurement of observer agreement for categorical data. *Biometrics* 159–174, 1977. 843571

[24] Langelier CA, and Connell JD (2005) Emotions and learning: Where brain based research and cognitive-behavioral counseling strategies meet the road. Rivier College Online Academic Journal 1(1):1–13.

[25] Larson, L.M., and Daniels, J.A., Review of the counseling self-efficacy literature. *Couns. Psychol.* 26(2):179–218, 1998.

[26] Larson, L.M., Suzuki, L.A., Gillespie, K.N., et al., Development and validation of the counseling self-estimate inventory. *J. Couns. Psychol.* 39(1):105, 1992.

[27] Lie, H., *Design and evaluation of a bdi-based virtual patient for the education of shared decision making*. Master’s thesis, Delft University of Technology, 2018.

[28] Lu, D., *Emotion model for child helpline training tool*. Master’s thesis, Delft University of Technology, 2003.

[29] Maier HW (2002) Role playing: structures and educational objectives. The International Child and Youth Care Network 36.

[30] Mascarenhas, S., Dias, J., Prada, R., et al., A dimensional model for cultural behavior in virtual agents. *Appl. Artif. Intell.* 24(6):552–574, 2010.

[31] McHugh, M.L., Interrater reliability: The kappa statistic. *Biochemia Med. *22(3):276–282, 2012. PMC3900052 23092060

[32] Van der Meij, H., Principles and heuristics for designing minimalist instruction. *Techn. Commun.* 42(2):243–261, 1995.

[33] Nissen-Lie, H.A., Rønnestad, M.H., Høglend, P.A., et al., Love yourself as a person, doubt yourself as a therapist? *Clin. Psychol. Psychother.* 24(1):48–60, 2017. 10.1002/cpp.1977 26450342

[34] Pereira, D., Oliveira, E., Moreira, N., et al., Towards an architecture for emotional bdi agents. In: *2005 portuguese conference on artificial intelligence*, IEEE, pp 40–46, 2005.

[35] Rogers, L., Developing simulations in multi-user virtual environments to enhance healthcare education. *British J. Educ. Technol.* 42(4):608–615, 2011.

[36] Salmi, S., Mérelle, S., Gilissen, R., et al., Content-based recommender support system for counselors in a suicide prevention chat helpline: Design and evaluation study. *J. Med. Int. Res.* 23(1):e21690, 2021. 10.2196/21690 PMC7819775 33410755

[37] Shorey, S., Ang, E., Yap, J., et al., A virtual counseling application using artificial intelligence for communication skills training in nursing education: Development study. *J. Med. Int. Res.* 21(10), 2019.10.2196/14658. 10.2196/14658 PMC6913997 31663857

[38] Sindahl, T.N.,* Chat Counselling for Children and Youth - A Handbook*, 2011.

[39] Sirocki, J., *Design and evaluation of a conversational agent model based on stance and bdi providing situated learning for triage-psychologists in the helpline of 113 suicide prevention*. Master’s thesis, Delft University of Technology, 2019.

[40] Stichting De Kindertelefoon, and De Kindertelefoon Jaarverslag 2020, 2020. https://jaarverslag.kindertelefoon.nl/2020, Accessed 2-May-2022.

[41] Wever, D., Hermens, H., and Vollenbroek-Hutten, M., Differences in use of a exercise-based tele-rehabilitation service delivered as substitute of or supplement to conventional care. In: *Sixth International Symposium on E-Health Services and Technologies*, pp 44–51, 2012.

